# MicroRNA-23a-3p influences the molecular mechanism of gastric cancer cells via CCL22/PI3K/Akt axis

**DOI:** 10.1080/21655979.2021.2002620

**Published:** 2021-12-07

**Authors:** Zhipeng Jiang, Min Su, Hua Chen, Limian Wu, Xinpei Yu, Zichuan Liu

**Affiliations:** a Department of Gastrointestinal Surgery, The Sixth Affiliated Hospital of Sun Yat-sen University, Guangzhou City, Guangdong Province, China; b Department of Internal Medicine, Guangzhou Development District Hospital, Guangzhou City, Guangdong Province, 510730, China; c Department of Oncology, Maoming people’s Hospital, Maoming City, Guangdong Province, 525000, China; d Department of Respiratory Medicine, Guangzhou Medical University, Guangzhou Institute of Respiratory Health, the First Affiliated Hospital of Guangzhou Medical University, Guangzhou City, Guangdong Province, China; e Deparment of Medical Oncology, Affiliated Cancer Hospital & Institute of Guangzhou Medical University, Guangzhou City, Guangdong Province, China; f Department of Health Ward, Afnfiliated Cancer Hospital & Institute of Guangzhou Medical University; GuangzhouHigh-LevelClinical KeySpecialtyConstructionProject; (2019-2021); ClinicalKeySpecialty ConstructionProject of Guangzhou Medical University; (YYPT202017), Guangzhou City, Guangdong Province, China; g Internal Medicine Section 2, Affiliated Cancer Hospital & Institute of Guangzhou Medical University, Guangzhou City, Guangdong Province, China; h Department of Cardiology, Beijing Chao-Yang Hospital, Beijing, China

**Keywords:** miR-23a-3p, gastric cancer, PI3K/AKT, molecular mechanism

## Abstract

A great many microRNAs (miRNAs) have been reported to play different roles in human cancers, including gastric cancer (GC). However, the specific character of miR-23a-3p in GC has not been elucidated. This study was to explore the function of miR-23a-3p in GC. The results manifested that miR-23a-3p was down-regulated in GC and patients with reduced miR-23a-3p had poor prognosis. Functional experiments assured that elevated miR-23a-3p refrained GC proliferation, invasion, migration, PIK3/Akt phosphorylation and apoptosis, while knockdown miR-23a-3p accelerated the growth of GC. Double luciferase report experiments manifested that miR-23a-3p targeted CCL22 expression. Functional rescue experiments affirmed that the repression of elevated miR-23a-3p on GC was reversed by simultaneous augmented CCL22. *In vivo*, elevated miR-23a-3p restrained the volume and tumor of GC and reduced the expression of CCL22 and phosphorylated PIK3/Akt, while knockdown miR-23a-3p motivated tumor growth. In conclusion, the results of this study indicate that miR-23a-3p plays a repressive role in GC, and affects the progression of GC via down-regulating CCL22 and blocking PI3K/AKT signal transduction pathway, which may offer a new molecular target for clinical treatment of GC.

## 1. Background

Gastric cancer (GC) is a familiar and frequently occurring malignant gastrointestinal tumor, threatening the safety of human life. Meanwhile 980,000 novel cases and 730,000 deaths exist in the world each year, ranking the third in both morbidity and mortality of malignancies [1]. In China, GC’s morbidity and mortality rank first and second among malignancies, or malignancies of the digestive system, and the mortality accounts for 23.18% of all malignancies [2]. There are some main approaches for clinical diagnosis of GC, including Ultrasound Computed Tomography, Magnetic Resonance Imaging and exclude-cytology. Nevertheless, there are still certain defects in the early diagnosis of GC by gas [3]. Screening indicators with lack of specificity and sensitivity is the most crucial element influencing the early diagnosis of GC, leading to the late stages of clinical diagnosis for the majority of GC patients, and ultimately unpleasing clinical efficacy and prognosis [4]. Therefore, the momentous, theoretical and direct guiding meanings were present in the study, exposing that the study of the molecular mechanism of the GC progression provides a basis for the selection of therapy scheme and prognosis prediction of molecular typing for early diagnosis of GC.

In the past few decades, t he functional studies of microRNAs (miRNAs) have affirmed a hopeful direction for the exploration of GC’s pathogenesis and the search for brand-new diagnostic markers and therapeutic targets [5,6]. A single-stranded non-coding small RNA molecule controls gene via either directly degrading mRNAs or interaction with target mRNAs [7,8]. MiRNAs were discovered in 1993, then the close association of the changes of miRNAs with tumors, including GC has been manifested [9]. A great many miRNAs, considered as tumor suppressor genes or oncogenes, have been testified to be crucial in the occurrence and development of tumors. Currently, abnormally modulated miRNAs in GC are not only implicated with the proliferation, the apoptosis and migration of GC cells, but also cause the change for the sensitivity to radiotherapy and chemotherapy via modulating kinds of tumor-related target genes [10–12]. For above reasons, insightful study of the function of miRNAs with their target genes in GC is beneficial for elucidating the pathogenesis, affirming potential therapeutic targets. It has also been manifested that miR-23a-3p is dysregulated in various tumors, containing laryngeal cancer, glioma and other cancers [13–15]. Recently, a study has reported that toosendanin induces apoptosis of MKN-45 human GC cells via miR-23a-3p /BCL2 axis [16].However, the biological function of miR-23a-3p in GC has not been fully elucidated.

In this study, it was speculated that miR-23a-3p might be involved in the proliferation, invasion, migration and EMT processes of GC, and this conjecture was confirmed through functional loss and acquisition experiments. It was also found that miR-23a-3p affected the activation of PI3K/Akt pathway in GC through targeted modulation of CCL22 expression.

## 2. Materials and methods

### 2.1. Clinical tissues

From May 2017 to January 2019, 84 GC and para-cancerous normal tissues were collected from The Sixth Affiliated Hospital of Sun Yat-sen University (Guangzhou, China). Immediately after surgical resection, the treatment of tissue samples was implemented with liquid nitrogen with the storage at −80°C. No reception of any radiation or chemotherapy in all GC patients was affirmed before surgery. The approval of experimental procedures has been conducted by the Ethics Committee of The Sixth Affiliated Hospital of Sun Yat-sen University. All enrolled patients had obtained written informed consent.

### 2.2. Cell culture

The introduction of GC cell lines MGC-803, BGC-823, SGC-7901, MKN-45 and MKN-7 and gastric mucosal epithelial cell GES-1 (American Type Culture Collection, MANASAS, VA) was carried out with RPMI-1640 (GE Healthcare Life Sciences, Logan, UT, USA) containing 1% penicillin/streptomycin (SolarBio, Beijing, China) and 10% fetal bovine serum (Gibco, Rockville, MD).

### 2.3. Cell transfection

The introduction of miR-23a-3p mimic (5ʹ-CCUUUAGGGACCGUUACACUA-3ʹ) or inhibitor (5ʹ-UAGUGUAACGGUCCCUAAAGG-3ʹ) and its negative control (mimic NC, 5ʹ-CGAGCUCACUGGACAACGCCG-3ʹ and inhibitor NC, 5ʹ-AGCUUAAGACAUUCCGAGGAAU-3ʹ)(all GenePharma, Shanghai, China), the siRNA targeting CCL22 (si-CCL22, sense 5ʹ-CCUGGGUGAAGAUGAUUCUCAAUAA-3ʹ and antisense 5ʹ-UUAUUGAGAAUCAUCUUCACCCAGG-3ʹ) and si-NC and CCL22 elevation vector (pcDNA 3.1-CCL22) and NC (pcDNA 3.1) (all Genechem, Shanghai, China) was implemented into the cells with liposomes 2000 (Invitgen) with according manufacturing instructions. Except miR-23a-3p inhibitor concentration of 100 nM, the transfection concentration of oligonucleotides or plasmids was 50 nM. After 48 h, the collection of transfected cells was required for follow-up experiments.

### 2.4. RNA extraction and Reverse transcription quantitative polymerase chain reaction (RT-qPCR)

RT-qPCR was performed as described above [17]. The extraction of total RNA from tissues and cells was implemented via Trizol ®reagents (InvitGen; Thermo Fisher Science, Inc.). The synthesis of the cDNA was carried out via PrimeScript reverse transcription kit (Qiyuan China Co., Ltd.) and conduction of RT-qPCR was with SYBR®Premix Extaq ™ (Baocara, Dalian, China) on the StepOnePlus™ real-time PCR system (Applied Biosystems, Foster City, California, USA). U6 or glyceraldehyde-3-phosphate dehydrogenase (GAPDH) was applied as an internal control for miRNA and mRNA separately. The quantification of miR-23a-3p and CCL22 was via 2^−ΔΔCq^. The primer sequences were as follows: miR-23a-3p: F: 5ʹ-ATCACATTGCCAGGGATTTCC-3ʹ; U6: F: 5ʹ-CTCGCTTCGGCAGCACA-3 ‘; GAPDH: F: 5ʹ -GGGAGCCAAAGGGTCATCA-3, R: 5ʹ-AGTGATGGCATGGACTGTGG-3ʹ; CCL22: F: 5´ -AGGTATGGTGCCAATGT-3 ´, R: 5´-CGGCAGGATTTTGAGGTCCA-3´.

### 2.5. Cell counting kit (CCK)-8 assay

There was determination of the survival rate of transfected GC cells via CCK-8 colorimetry [18], culture in 1 × 10^4^ density in 96-well plates for 0, 24, 48, 72 and 96 h, and incubation with 10 μL CCK-8 reagent in each well. The measurement of the absorbance at 490 nm was conducted via a microplate analyzer.

### 2.6. Flow cytometry

Apoptosis was detected as previously shown [19]. There was suspension of GC cells in 500 µL binding buffer, staining with 5 µL Annexin V-enhanced green fluorescent protein (EGFP) and 5 µL propidium iodide (PI) in darkness, and calculation of apoptosis rate via flow cytometry (BD Biosciences).

### 2.7. Transwell assay

For migration assay, the seeding of introduced cells (0.5 × 10^6^ cells/mL) was carried out at the chamber with membrane (Corning Costar Corp., Cambridge, MA, USA). Then fixation of the cells crossing the membrane, staining with hematoxylin, and counting were required. The quantification of cell migration was exposed through the number of cells per sample crossing the well from 5 different randomly selected field of view under the microscope (× 100) [20]. For invasion analysis, the incubation of Matrigel (BD Biosciences) diluted to 1 mg/mL in serum-free cold cell medium in the chamber was implemented until the solidification of matrix gel.

### 2.8. Western blot

The harvest of cells and lysis in lysate buffers containing phenylmethylsulfonyl fluoride, protease inhibitors, and phosphatase inhibitors were executed. The determination of protein concentration via the bicinchoninic acid (BCA) Protein Assay Kit (Thermo Science, USA), separation on sulfate polyacrylamide gel electropheresis and electroblot onto a nitrocellulose membrane (Bio-Rad, Hercules, USA) were affirmed. There was block of membrane with 5% bovine serum albumin and incubation with the antibodies: E-cadherin (3195), snail (3879), p-PI3K (4228), PI3K (4257), Akt (9272), p-Akt (4060) (all Cell Signaling Technology), N-cadherin (ab18203), Bax (ab32503), Bcl-2 (ab32124), GAPDH (ab8245), CCL22 (ab9847) (all Abcam). Then, the wash of membrane was required with fluorescence coupled secondary antibody and detection of the protein via infrared dichroic laser scanning imaging system (Li-COR, Oderson sey).

### 2.9. The luciferase reporter assay

Inserting of MUT or WT-CCL22-3ʹUTR was implemented into the pmirGlo luciferase reporter vector (Promega Corporation) [21]. The introduction of HEK293T cells was conducted with luciferase vector and miR-23a-3p mimic or its NC with Lipofetamine®2000 (Invitgen; Thermo Fisher Science, Inc.), and the luciferase activity’s examination was via a dual luciferase assay system (Promega).

### 2.10. Xenografted tumors in nude mice

The animal testing procedures were based on the ‘Guidelines for the Care and Use of Laboratory Animals’ published by the National Institutes of Health. The approvement of the study was conducted by the Ethics Committee of The Sixth Affiliated Hospital of Sun Yat-sen University Central Hospital. The male nude mice (BALB/c, 4 weeks of age, body weight 20 ± 2 g) [Shanghai Experimental Animal Center, Chinese Academy of Sciences (Shanghai, China)] were randomly assigned into 4 groups (n = 6): the mimic NC, the miR-23a-3p mimic, the inhibitor NC, and the miR-23a-3p inhibitor. The injection of transfected MGC-803 cells 0.2 mL (1 × 10^7^) in logarithmic growth phase was implemented into the left intradermal axilla, and the measurement of maximum diameter (L) and minimum diameter (W) of the xenografted tumor was via a vernier caliper, and the volume was calculated every other week after injection. Volume = (L × W)^2^ × 0.5. At the end of four weeks, the anesthesia of mice was via chloral hydrate, with euthanasia via decollation. The weighing of isolated tumors and fixation in 4% paraformaldehyde were carried out.

### 2.11. Immunohistochemistry

There was embedding of the tumor tissues with paraffin wax, conventional dewaxing, hydration, antigen repair, and dripping with hydrogen peroxide. Then the incubation of sections with CCL22 (ab9847, Abcam), dripping with right dose of the secondary antibody, staining with Diaminobenzidine solution, counterstaining with hematoxylin were required.

### 2.12. Statistical analysis

All data were presented as mean ± standard deviation (SD) and analyzed with SPSS 19.0 software. Two-group comparison was conducted via student’s t-test, and the comparison among multiple groups was via one-way analysis of variance (ANOVA). Correlation was explored via Pearson correlation coefficient analysis. *P* < 0.05 was considered statistically significant.

## 3. Results

### 3.1. MiR-23a-3p in GC is downregulated and associated with poor prognosis

**Figure 1.**
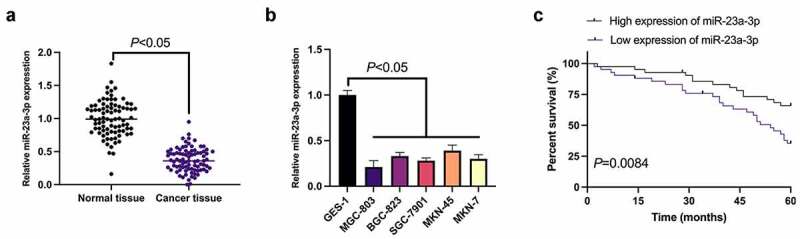
Declined miR-23a-3p is present in GC and connected with poor prognosis. (a). The detection of miR-23a-3p in GC tissues and adjacent normal tissues via RT-qPCR; (b). The detection of miR-23a-3p in GC cell lines (MGC-803, BGC-823, SGC-7901, MKN-45, MKN-7) and gastric mucosa epithelial cells GES-1 via RT-qPCR; (c). The connection of miR-23a-3p with survival prognosis in GC patients; The values were shown as mean ± SD (B, n = 3); Survival prognosis of the two groups was compared via Log-rank (Mantel-Cox) text

MiR-23a-3p was first examined in GC (Figure 1a, b), manifesting that miR-23a-3p in GC tissues and cell lines was obviously reduced versus adjacent normal tissues and gastric mucosal epithelial cells GES-1. Since miR-23a-3p was the lowest in MGC-803 cells, MGC-803 cells were then chosen for subsequent functional verification tests. Meanwhile, the survival prognosis graph exposed a worse survival prognosis in GC patients with knockdown miR-23a-3p (Figure 1c). According to the median expression, GC patients were divided into the miR-23a-3p elevation and reduction groups. In Table 1, it was clarified that the expression of miR-23a-3p was linked with the distal metastasis and TNM staging of GC patients. Briefly, declined miR-23a-3p was present in GC and implicated with unpleasing prognosis.

**Table 1.** Correlation of miR-23a-3p with clinicopathological features

			MiR-23a-3p expression		*P*
Features	Groups	n	elevation (n = 42)	reduction (n = 42)	
Age	< 60 years	28	12	16	0.3545
	60 years or more	56	30	26	
Gender	male	36	21	15	0.1859
	female	38	21	27	
Tumor size (cm)	< 5 cm	17	10	7	0.4152
	5 cm or more	67	32	35	
Distant metastases	Yes	49	12	37	< 0.0001
	No	35	30	5	
TNM staging	I+ II	25	19	6	0.0019
	III+IV	59	23	36	
Lymphatic metastasis	Yes	62	33	29	0.3209
	No	22	9	13	

The correlation between miR-23a-3p and pathological features of patients was detected via Pearson correlation analysis.

### 3.2. MiR-23a-3p constrains GC cell survival and metastasis

**Figure 2.**
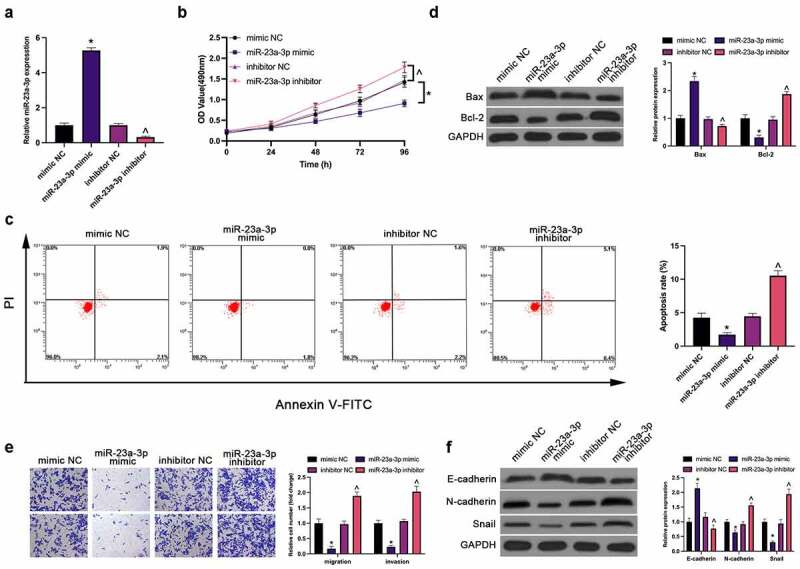
MiR-23a-3p represses GC cell survival and metastasis. (a). The detection of miR-23a-3p via RT-qPCR; (b). The detection of cell proliferation via CCK-8; (c). The detection of cell apoptosis rate via Flow cytometry; (d). The detection of apoptosis-linked proteins Bax and Bcl-2 via Western blot; (e). The detection of cell invasion and migration via Transwell; (f). The detection of EMT-related proteins E-cadherin, N-cadherin and Snail via Western blot; In MGC-803 cells after transfection with miR-23a-3p mimic/inhibitor. The values were shown as mean ± SD (n = 3); Vs the mimic NC, *P < 0.05; Vs the inhibitor NC, ^ *P* < 0.05

Next, treatment of miR-23a-3p was conducted in MGC-803 cells by transfection (Figure 2a). Plenty of assays have manifested the repression of MGC-803 cell proliferation, invasion, migration, N-cadherin and snail and facilitation of apoptosis (elevated apoptosis rate and Bax, and declined Bcl-2) and E-cadherin (EMT) via elevation of miR-23a-3p, which was reversed via knockdown of it (Figure 2b-f).

### 3.3. MiR-23a-3p directly targets CCL22

**Figure 3.**
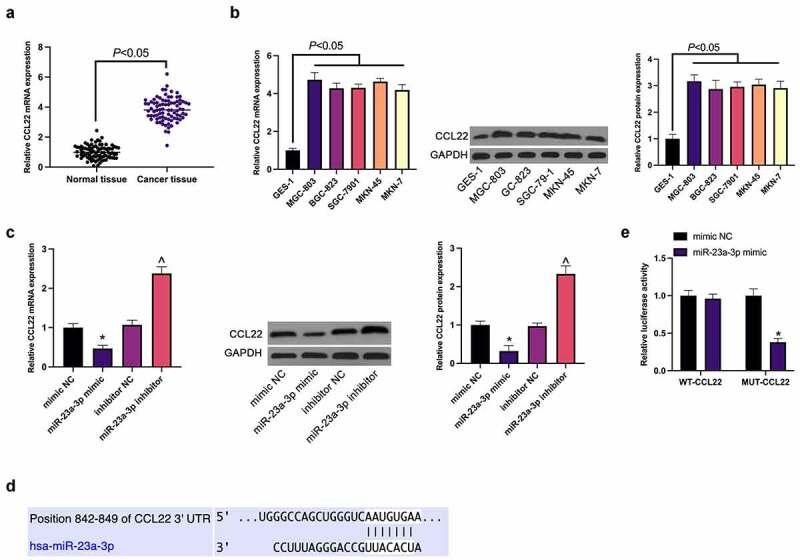
MiR-23a-3p directly targets CCL22. (a). The detection of CCL22 in GC and adjacent normal tissues via RT-qPCR; (b). The detection of CCL22 in GC cell lines (MGC-803, BGC-823, SGC-7901, MKN-45, MKN-7) and gastric mucosa epithelial cells GES-1 via RT-qPCR and Western blot; (c). The detection of CCL22 in MGC-803 cells transfected with miR-23a-3p mimic/inhibitor via RT-qPCR and Western blot; (d). The potential binding sites of miR-23a-3p and CCL22 predicted via TargetScan database (http://www.targetscan.org); (e). The targeting of miR-23a-3p with CCL22 verified via the luciferase report assay; The values were shown as mean ± SD (n = 3); Vs the mimic NC, *P < 0.05; Vs the inhibitor NC, ^ *P* < 0.05

Subsequently, the potential target gene of miR-23a-3p was explored. The elevation of CCL22 was affirmed in GC, consistent with the results (Figure 3a, b). Meanwhile, the suppression or promotion of CCL22 in MGC-803 cells was exposed via up-regulation or down-regulation of miR-23a-3p separately (Figure 3c). Then via TargetScan database was predicted the existence of potential combination of loci of miR-23a-3p with CCL22 (Figure 3d), which was confirmed by luciferase report analysis (Figure 3e), manifesting as a decrease of luciferase activity in the miR-23a-3p mimic via WT-CCL22-3ʹUTR, and no effect via MUT-CCL22-3ʹUTR.

### 3.4. MiR-23a-3p restrains GC progression via targeting CCL22

**Figure 4.**
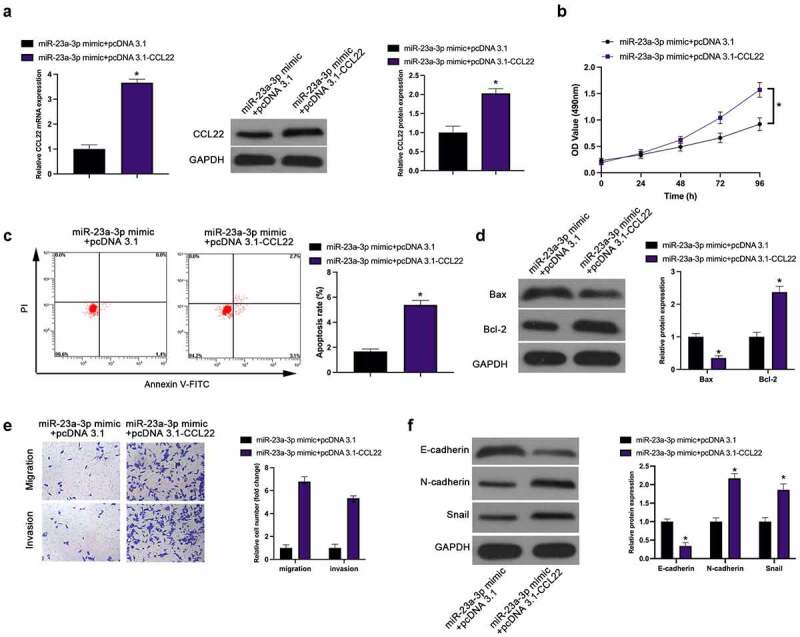
MiR-23a-3p suppresses GC progression via targeting CCL22. (a). The detection of CCL22 via RT-qPCR and Western blot; (b). The detection of cell proliferation via CCK-8; (c). The detection of cell apoptosis rate via Flow cytometry; (d). The detection of Bax and Bcl-2 via Western blot; (e). The detection of cell invasion and migration via Transwell; (f). The detection of EMT-related proteins E-cadherin, N-cadherin and Snail via Western blot; In MGC-803 cells after transfection with miR-23a-3p mimic + pcDNA 3.1-CCL22. The values were shown as mean ± SD (n = 3); Vs the miR-23a-3p mimic + pcDNA 3.1, *P < 0.05

For investigation of the interaction of CCL22 with miR-23a-3p, the transfection of miR-23a-3p mimic and pcDNA 3.1-CCL22 was implemented into MGC-803 cells, affirming the restoration of, reduced CCL22 induced via miR-23a-3p mimic, via pcDNA 3.1-CCL22 (Figure 4a). Functionally, elevated CCL22 turned around the repression of miR-23a-3p mimic on MGC-803 cell proliferation, invasion, migration and EMT, as well as the promotion of apoptosis rate (Figure 4b-f). Briefly, up-regulation of CCL22 weakens the suppressive effect of miR-23a-3p on GC.

### 3.5. MiR-23a-3p restrains the PI3K/Akt pathway in GC through CCL22

**Figure 5.**
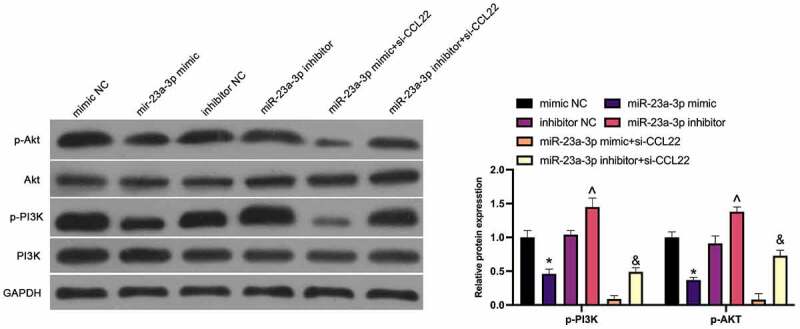
MiR-23a-3p depresses the PI3K/Akt pathway in GC through CCL22. The effect of miR-23a-3p and CCL22 on PI3K/AKT pathway protein phosphorylation in GC detected by Western blot. The values were shown as mean ± SD (n = 3); Vs the mimic NC, *P < 0.05; Vs the inhibitor NC, ^*P* < 0.05; Vs the miR-23a-3p mimic + si-CCL22, ^&^*P* < 0.05

For further clarification of the molecular mechanism of miR-23a-3p in GC, the modulation of miR-23a-3p on the PI3K/AKT pathway (PI3K, p-PI3K, AKT, p-AKT) was studied in MGC-803 cells (Figure 5), exposing the repression of the phosphorylation of PI3K and Akt via elevation of miR-23a-3p, while silencing of which manifested a promoted effect. The suppressive PI3K and Akt phosphorylation via miR-23a-3p mimic and the facilitated one via miR-23a-3p inhibitor were enhanced and weakened separately via si-CCL22. Shortly, miR-23a-3p strengthening blocks the PI3K/AKT pathway in GC.

### 3.6. *MiR-23a-3p restrains GC cell growth* in vivo

**Figure 6.**
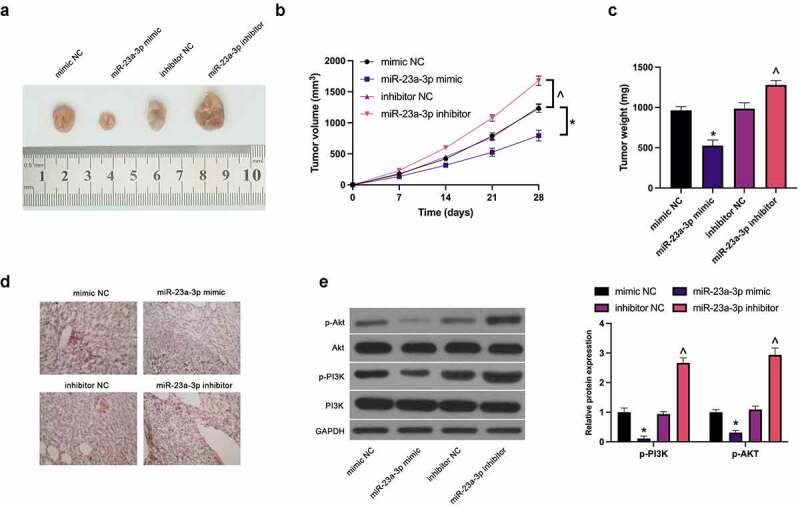
MiR-23a-3p restrains GC cell growth *in vivo.* (a). Representative pictures of the tumor; (b). The tumor volume; (c). The weight of the tumor; (d). The detection of CCL22 in tumor via immunohistochemistry; (e). The detection of phosphorylation of PI3K/Akt in tumor via Western blot. The values were shown as mean ± SD (n = 3); Vs the mimic NC, *P < 0.05; Vs the inhibitor NC, ^*P* < 0.05

For verification of the function of miR-23a-3p on GC cells *in vivo*, MGC-803 cells transfected with miR-23a-3p mimic and miR-23a-3p inhibitor were implanted into nude mice, affirming the apparently reduced tumor volume and weight via elevated miR-23a-3p (Figure 6a-c), while silencing of miR-23a-3p accelerated tumor growth. Immunohistochemistry manifested the separate cutback and strengthening of CCL22 in tumors via enhancement or silencing of miR-23a-3p (Figure 6d). Moreover, there was the downturn of PI3K/Akt phosphorylation in tumors via up-regulation of miR-23a-3p, while loss of which caused the augment (Figure 6e). Briefly, miR-23a-3p depresses GC growth *in vitro* through the PI3K/Akt pathway mediated by CCL22.

## 4. Discussion

GC is a familiar cancer worldwide, inducing severe death, with only a 20% survival rate for existing treatments. Most patients were identified as advanced or metastatic GC at the time of initial diagnosis, with less than 15% 5-year survival rate of patients with advanced GC [16]. It is vital of the emergence of new therapeutic targets for improvement of the early diagnosis and survival of GC. In this study, it was exposed that miR-23a-3p, a brand-new target of GC, is supposed to repress GC progression via strengthening of CCL22 and downturn of the PI3K/AKT pathway.

The link of miRNAs with cancer stems from Carlin’s work on chronic lymphoblastic leukemia, triggering the crazy research on miRNA with cancer [17]. Alleles deletion of miR-15a and −16 is manifested in patients with chronic lymphocytic leukemia, meanwhile, miRNAs are closely implicated with the onset of tumors, including GC [18–20]. Previous studies have exposed the crucial miR-23a-3p in various malignancies’ occurrence and the application of the miR as a marker for the diagnosis [13–15]. However, miR-23a-3p in GC is hardly explored. For further understanding of the possible part of miR-23a-3p in GC’s occurrence and development, the detection of miR-23a-3p in human GC tissues and cell lines was conducted, ensuring the downturn of miR-23a-3p in GC, further implicating the repressive effect in GC progression.

Nowadays, some tumor markers are applied for auxiliary diagnosis, such as CA-125, AFP, CEA, etc. Nevertheless, the sensitivity and specificity of them are not abundant for GC’s early diagnosis [21]. An evading digestion of ribonuclease via some serum miRNAs was demonstrated for their stability. Additionally, there were obvious differences of miRNAs expression in serum of some cancer patients with normal people, exposing the role of specific tumor markers. For instance, strengthening miR-21, miR-155 and miR-210 are assured in serum of B-cell lymphoma patients [22]. There is elevated miR-141 in serum of 25 prostate cancer patients, which is correlated with prostate-specific antigen. Therefore, miRNAs have great application potential as tumor markers in diseases’ diagnosis [23]. MiRNAs have been found to be vital in cell apoptosis, invasion, metastasis, differentiation, and proliferation [24]. MiRNAs mainly participate in the differentiation of cardiac muscle, nervous system, hematopoietic cells and skeletal muscle. Malpeli *et al*. identified 8 miRNAs, including miR-323, −138, −9, −211, and −149, etc, which are implicated with B cells’ late differentiation [25]. With the in-depth research on the relationship of miRNAs with tumors, the abnormal miRNAs in tumors are a crucial molecule controlling tumor cell growth. Among which, most miRNAs repress cell proliferation or induce apoptosis, manifesting the tumor depressive influence, while a few exist as oncogenes via promotion of cell proliferation and repression of apoptosis [26–28]. For these reasons, miRNAs are supposed to be essential in the biological behavior of cancer cells via modulation of related transcription factors, thereby facilitating or restraining the cancer progression. So far, the studies on the correlation of miR-23a-3p with tumors are relatively few, so the study on the function of miR-23a-3p in tumors is still original. Therefore, *in vitro* experiments on GC cell progression were conducted, exposing the downturn via strengthening miR-23a-3p, which is considered a tumor suppressor gene in GC’s development. In the meantime, the induction of GC apoptosis manifested as repression of Bcl-2 and enhancement of Bax was implemented via miR-23a-3p. Moreover, the knockdown of EMT and PI3K/Akt pathways in GC cells was manifested via miR-23a-3p elevation. Previous study has confirmed the up-regulation of CCL22 in GC and facilitation on GC progression [29]. In this study, it was also assured the elevation of CCL22 in GC, and miR-23a-3p could depress GC’s progression via down-regulation of CCL22 and blocking of PI3K/Akt pathway. CCL22 is a kind of chemokine. A previous study clarifies that M2 macrophage-derived CCL22 can motivate the migration ability of hepatocellular carcinoma and EMT behavior [22]. Another study manifests that curcumin reduces EMT potential in squamous cell carcinoma of the neck, which is linked with declined CCL22 [23]. In this study, it was found that knockdown CCL22 apparently repressed EMT capacity in GC. A former study has clarified that CCL22 can induce phosphorylation of PI3K/Akt [24,25], which is beneficial to accelerate EMT behavior of GC. In this study, knockdown CCL22 prevented the phosphorylation of PI3K/Akt in GC cells, which might be directly related to the reduction of EMT potential.

The limitations of this study are as follows: 1. The expression of miR-23a-3p in serum of GC patients is ambiguous; 2. Only one kind of GC cell was studied, and it is not clear whether miR-23a-3p/CCL22 axis has the same impacts on other GC cell lines; 3. Only the influence of miR-23a-3p/CCL22 axis on the growth of GC tumor *in vivo* was observed, but the effect on the distal metastasis of GC was not clear; 4. MiR-23a-3p/CCL22 axis knockdown or overexpression was not conducted in patients with GC.

## 5. Conclusion

All in all, the results of this study suggest that miR-23a-3p is down-regulated in GC, and linked with the poor prognosis of GC patients. In addition, miR-23a-3p restrains the survival and metastasis of GC cells via targeting CCL22, and is thought to block EMT in GC and inactivate the PI3K/Akt pathway.

## References

[1] Bray F, Ferlay J, Soerjomataram I, et al. Global cancer statistics 2018: GLOBOCAN estimates of incidence and mortality worldwide for 36 cancers in 185 countries. CA Cancer J Clin. 2018;68(6):394–424. 30207593 10.3322/caac.21492

[2] Zong L, Abe M, Seto Y, et al. The challenge of screening for early gastric cancer in China. Lancet. 2016;388(10060):2606. 27894662 10.1016/S0140-6736(16)32226-7

[3] Pasechnikov V, Chukov S, Fedorov E, et al. Gastric cancer: prevention, screening and early diagnosis. World J Gastroenterol. 2014;20(38):13842–13862. 25320521 10.3748/wjg.v20.i38.13842 PMC4194567

[4] Brungs D, Aghmesheh M, Vine KL, et al. Gastric cancer stem cells: evidence, potential markers, and clinical implications. J Gastroenterol. 2016;51(4):313–326. 26428661 10.1007/s00535-015-1125-5

[5] Liu HS, Xiao HS. MicroRNAs as potential biomarkers for gastric cancer. World J Gastroenterol. 2014;20(34):12007–12017. 25232237 10.3748/wjg.v20.i34.12007 PMC4161788

[6] Al-Qatati A, Akrong C, Stevic I, et al. Plasma microRNA signature is associated with risk stratification in prostate cancer patients. Int J Cancer. 2017;141(6):1231–1239. 28571116 10.1002/ijc.30815

[7] Haendeler J, Mlynek A, Büchner N, et al. Two isoforms of sister-of-mammalian grainyhead have opposing functions in endothelial cellsand in vivo. Arterioscler Thromb Vasc Biol. 2013;33(7):1639–1646. 23685552 10.1161/ATVBAHA.113.301428

[8] Falcone G, Felsani A, D’Agnano I. Signaling by exosomal microRNAs in cancer. J Exp Clin Cancer Res. 2015;34(1):32. 25886763 10.1186/s13046-015-0148-3 PMC4391656

[9] Mirzaei H, Khataminfar S, Mohammadparast S, et al. Circulating microRNAs as potential diagnostic biomarkers and therapeutic targets in gastric cancer: current status and future perspectives. Curr Med Chem. 2016;23(36):4135–4150. 27538692 10.2174/0929867323666160818093854

[10] Yang T-S, Yang X-H, Wang X-D, et al. MiR-214 regulate gastric cancer cell proliferation, migration and invasion by targeting PTEN. Cancer Cell Int. 2013;13(1):68. 23834902 10.1186/1475-2867-13-68 PMC3716801

[11] Wang F, Li T, Zhang B, et al. MicroRNA-19a/b regulates multidrug resistance in human gastric cancer cells by targeting PTEN. Biochem Biophys Res Commun. 2013;434(3):688–694. 23603256 10.1016/j.bbrc.2013.04.010

[12] Hsu K-W, Wang A-M, Ping Y-H, et al. Downregulation of tumor suppressor MBP-1 by microRNA-363 in gastric carcinogenesis. Carcinogenesis. 2014;35(1):208–217. 23975832 10.1093/carcin/bgt285

[13] Zhang XW, Liu N, Chen S, et al. Upregulation of microRNA-23a regulates proliferation and apoptosis by targeting APAF-1 in laryngeal carcinoma. Oncol Lett. 2015;10(1):410–416. 26171041 10.3892/ol.2015.3238 PMC4487154

[14] Liu N, Sun YY, Zhang XW, et al. Oncogenic miR-23a in pancreatic ductal adenocarcinogenesis via inhibiting APAF1. Dig Dis Sci. 2015;60(7):2000–2008. 25701323 10.1007/s10620-015-3588-x

[15] Hu X, Chen D, Cui Y, et al. Targeting microRNA-23a to inhibit glioma cell invasion via HOXD10. Sci Rep. 2013;3:3423. 24305689 10.1038/srep03423 PMC3851882

[16] Shao S, Li S, Liu C, et al. Toosendanin induces apoptosis of MKN-45 human gastric cancer cells partly through miR-23a-3p-mediated downregulation of BCL2. Mol Med Rep. 2020 Sep;22(3):1793–1802. 32582989 10.3892/mmr.2020.11263 PMC7411345

[17] Wang S, Zhu W, Qiu J, et al. lncRNA SNHG4 promotes cell proliferation, migration, invasion and the epithelial-mesenchymal transition process via sponging miR-204-5p in gastric cancer. Mol Med Rep. 2021 Jan;23(1):85. 33236157 10.3892/mmr.2020.11724 PMC7716413

[18] Ma C, Wang X, Yang F, et al. Circular RNA hsa_circ_0004872 inhibits gastric cancer progression via the miR-224/Smad4/ADAR1 successive regulatory circuit. Mol Cancer. 2020 Nov 10;19(1):157. 33172486 10.1186/s12943-020-01268-5 PMC7654041

[19] Luo Y, Liang M, Yao W, et al. Functional role of lncRNA LOC101927497 in N-methyl-N’-nitro-N-nitrosoguanidine-induced malignantly transformed human gastric epithelial cells. Life Sci. 2018 Jan 15;193:93–103. 29223541 10.1016/j.lfs.2017.12.007

[20] Han X, Liu Z. Long non-coding RNA JPX promotes gastric cancer progression by regulating CXCR6 and autophagy via inhibiting miR-197. Mol Med Rep. 2021 Jan;23(1):60. 33215222 10.3892/mmr.2020.11698 PMC7723066

[21] Sun G, Li Z, He Z, et al. Circular RNA MCTP2 inhibits cisplatin resistance in gastric cancer by miR-99a-5p-mediated induction of MTMR3 expression. J Exp Clin Cancer Res. 2020 Nov 17;39(1):246. 33198772 10.1186/s13046-020-01758-w PMC7670601

[22] Yeung OW, Lo CM, Ling CC, et al. Alternatively activated (M2) macrophages promote tumour growth and invasiveness in hepatocellular carcinoma. J Hepatol. 2015 Mar;62(3):607–616. 25450711 10.1016/j.jhep.2014.10.029

[23] Kötting C, Hofmann L, Lotfi R, et al. Immune-stimulatory effects of curcumin on the tumor microenvironment in head and neck squamous cell carcinoma. Cancers (Basel). 2021 Mar 16;13(6):1335. 33809574 10.3390/cancers13061335 PMC8001767

[24] Katsuya H, Cook LBM, Rowan AG, et al. Phosphatidylinositol 3-kinase-δ (PI3K-δ) is a potential therapeutic target in adult T-cell leukemia-lymphoma. Biomark Res. 2018 Jul 18;6:24. 30034808 10.1186/s40364-018-0138-7 PMC6052569

[25] Wei C, Yang C, Wang S, et al. M2 macrophages confer resistance to 5-fluorouracil in colorectal cancer through the activation of CCL22/PI3K/AKT signaling. Onco Targets Ther. 2019 Apr 18;12:3051–3063. 31114248 10.2147/OTT.S198126 PMC6489624

